# Clinical efficacy of femtosecond laser for myopia: A systematic review protocol of randomized controlled trial

**DOI:** 10.1097/MD.0000000000017906

**Published:** 2019-11-22

**Authors:** Xiao-fang Wang, Jun-xia Zhang

**Affiliations:** a Department of Ophthalmology, Hanzhong 3201 Hospital, Hanzhong; b Department of Ophthalmology, Yan’an People's Hospital, Yan’an, Shaanxi, China.

**Keywords:** efficacy, femtosecond laser, myopia, safety

## Abstract

**Background:**

Femtosecond laser (FL) is an effective method to treat patients with myopia, but its relative efficacy and safety is still unclear. Thus, this study will be conducted to assess the efficacy and safety of FL for myopia systematically.

**Methods:**

This study will systematically retrieve the following electronic databases up to the present: Cochrane Library, PubMed, EMBASE, Web of Science, PsycINFO, Allied and Complementary Medicine Database, Chinese Biomedical Literature Database, Wanfang, VIP, and China National Knowledge Infrastructure. All electronic databases will be searched without any limitations of language and publication status. RevMan 5.3 software will be utilized for statistical analysis.

**Results:**

We will summarize the targeted results evaluating the efficacy and safety of FL for patients with myopia.

**Conclusions:**

This study will provide a comprehensive evidence summary on FL for patients with myopia.

PROSPERO registration number: PROSPERO CRD42019148659.

## 1 Introduction

Myopia is a very common and yet perplexing ocular vision-threatening disorder.^[1–3]^ Its prevalence is increasing around the world annually.^[4–6]^ Its complications are often associated with high costs.^[5,7–10]^ Previous studies have found that it can be traced back to the childhood of school age onset myopia.^[6,11,12]^ Thus, it is very important to control and treat myopia as early as possible.^[13–15]^ Although several treatments are responsible for its management, its mechanism is still poorly understood.^[16]^ Femtosecond laser (FL) is reported to treat myopia effectively and safety.^[17–26]^ However, its results are still inconsistent. Therefore, this study will aim to assess the efficacy and safety of FL for patients with myopia.

## 2 Methods

### 2.1 Eligibility criteria

#### 2.1.1 Types of studies

Randomized controlled trials (RCTs) assessing the efficacy and safety of FL for patients with myopia will be included. We will exclude nonclinical studies, noncontrolled trials, and non-RCTs.

#### 2.1.2 Types of interventions

In the experimental group, all patients received any forms of FL will be included.

In the control group, we will include all patients who have undergone any interventions, but not FL.

#### 2.1.3 Types of participants

Participants with clinical diagnosis of myopia will be considered for inclusion without any restrictions of race, sex, age, and their economic sources.

#### 2.1.4 Types of outcome measurements

The primary outcome is myopia progression, as assessed by cycloplegic autorefraction. The secondary outcomes are uncorrected distance visual acuity, corrected distance visual acuity, axial length, vision stability, refraction, contrast sensitivity, and adverse events.

### 2.2 Search strategy

#### 2.2.1 Electronic databases sources

We will systematically perform a literature records search from the following electronic databases up to the present: Cochrane Library, PubMed, EMBASE, Web of Science, PsycINFO, Allied and Complementary Medicine Database, Chinese Biomedical Literature Database, Wanfang, VIP, and China National Knowledge Infrastructure. All electronic databases will be searched with no limitations of language and publication status. The sample of search strategy for Cochrane Library is presented in Table 1. Similar search strategy for other electronic databases will be adapted.

**Table 1:**
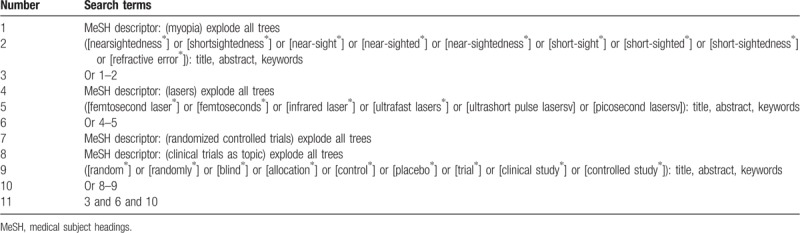
Search strategy utilized in Cochrane Library.

#### 2.2.2 Other literature sources

We will also search dissertations, ongoing trials, conference abstracts, and reference lists of relevant reviews.

### 2.3 Study selection

We will screen the titles and abstracts independently by 2 authors according to the eligibility criteria, and any irrelevant studies will be excluded. The remaining studies will be read for full-text assessment. Any divergences regarding the study selection between 2 authors will be solved by a third author through discussion. Details of study selection are shown in the flowchart.

### 2.4 Data extraction

All essential data information will be extracted by 2 independent authors from each eligible study using standard data extraction sheet. It comprises of the following information: title, year of publication, country, patient characteristics, study objective, study setting, study design, randomization, blinding, concealment, sample size, follow-up information, treatment details, comparators, outcome measurements, and safety. Any discrepancies of data extraction between 2 authors will be resolved by a third author.

### 2.5 Assessment of risk of bias

Cochrane risk of bias tool is utilized for methodological quality assessment for each eligible study according to the Cochrane Handbook for Systematic Review of Interventions. Two authors will independently assess the risk of bias, and any discrepancies will be reached by a third author via consensus.

### 2.6 Missing data management

If there are missing or incomplete data for the primary results, original corresponding authors will be contacted to require those data. If that information cannot be obtained, only available data will be performed.

### 2.7 Measurements of treatment effect

Continuous outcome data will be measured using mean difference or standardized mean difference and 95% confidence intervals. Dichotomous outcome data will be presented as risk ratio and 95% confidence intervals.

### 2.8 Assessment of heterogeneity

We will identify heterogeneity among eligible studies using *I*^2^ test. We defined that: *I*^2^ ≤ 50% is low heterogeneity, and a fixed-effect model will be applied; and *I*^2^ > 50% is substantial heterogeneity, and a random-effect model will be used.

### 2.9 Subgroup analysis

A subgroup analysis will be performed to identify the source of heterogeneity based on the different treatments, controls, and outcomes.

### 2.10 Sensitivity analysis

A sensitivity analysis will be conducted to find out the stability and robustness of outcome results by removing studies with high risk of bias.

### 2.11 Reporting bias

Funnel plot and Egger regression test^[27,28]^ will be carried out to check any publication bias if more than 10 eligible studies will be included.

### 2.12 Data synthesis

Statistical analysis will be performed using RevMan 5.3 software. We will synthesize the data for each outcome measurement with similar study design, participants, interventions, and comparators. If low heterogeneity (*I*^2^ ≤ 50%) exists, we will perform meta-analysis. Otherwise, if high heterogeneity (*I*^2^ > 50%) exists, we will conduct subgroup analysis. If there is still substantial heterogeneity after subgroup analysis, data pooling is deemed inappropriate, and we will report a qualitative discussion and a narrative description.

## 3 Discussion

At present, the therapeutic efficacy of FL on myopia is satisfactory. However, its efficacy of evidence-based medicine literature is still inconclusive. This study will systematically evaluate the efficacy and safety of FL for patients with myopia. The results of this study will summarize the latest evidence on assessing efficacy and safety of FL for myopia. They will also provide helpful evidence for patients, clinician, as well as for the policy-makers.

## Author contributions

**Conceptualization:** Xiao-fang Wang, Jun-xia Zhang.

**Data curation:** Xiao-fang Wang, Jun-xia Zhang.

**Formal analysis:** Xiao-fang Wang.

**Investigation:** Jun-xia Zhang.

**Methodology:** Xiao-fang Wang.

**Project administration:** Jun-xia Zhang.

**Resources:** Xiao-fang Wang.

**Software:** Xiao-fang Wang.

**Supervision:** Jun-xia Zhang.

**Validation:** Xiao-fang Wang, Jun-xia Zhang.

**Visualization:** Xiao-fang Wang, Jun-xia Zhang.

**Writing – original draft:** Xiao-fang Wang.

**Writing – review & editing:** Xiao-fang Wang, Jun-xia Zhang.
